# The Dispensers' Column

**Published:** 1888-06-23

**Authors:** 


### The Dispensers' Column.

It is estimated that one-half of all the drugs imported into the United States is consumed in the manufacture of patent medicines.

As the *American Druggist* truly states: We are dependent upon Zanzibar as the chief source of the supplies of cloves and their oil which come to our markets, and the existence there of an ad valorem duty of 30 per cent. —the principal source of the Sultan's revenue—makes it highly important that other sources of supply should be fostered. The Moluccas are, perhaps, the next most important producers of cloves, and the next to them are the Straits Settlements, Mauritius, and the Reunion. Cloves are remarkable for the large amount of essential oil which they contain, no official drug exceeding them in this respect. From 16 to 22 per cent. has generally been considered to be the range of the yield; but of late years Zanzibar cloves have stubbornly refused to give more than 17 per cent. The Mauritius cultivators have recently endeavoured to improve upon this by using the cloves in the green or undried condition, and the experiment has been attended with excellent results. A small consignment of the oil thus prepared has recently been received in London. In colour, odour, and taste this oil resembles ordinary oil of cloves; if anything, the odour is more fragrant, owing to the flower-buds being put into the still before they are dried. The oil has a specific gravity of 1·048 at 18 degs. C., is soluble in less than its own weight of rectified spirit, and conforms to the reliable chemical tests for oil of cloves. It therefore corresponds with the official requirements, and is entitled to become a commercial article.

